# Educational Inequalities and Obesity: Association and Attenuation After Lifestyle Adjustment in a Cross-Sectional Working-Age Population

**DOI:** 10.3390/medsci14030351

**Published:** 2026-06-27

**Authors:** María Teófila Vicente-Herrero, Pedro J. Tárraga López, Carla Busquets-Cortés, Lluis Rodas Cañellas, Ángel Arturo López-González, José Ignacio Ramírez-Manent

**Affiliations:** 1 ADEMA University School, University of the Balearic Islands, 07009 Palma, Spain; correoteo@gmail.com (M.T.V.-H.); ll.rodas@eua.edu.es (L.R.C.); a.lopez@eua.edu.es (Á.A.L.-G.); joseignacio.ramirez@ibsalut.es (J.I.R.-M.); 2 Faculty of Medicine, University of Castilla La Mancha, 02008 Albacete, Spain

**Keywords:** obesity, socioeconomic factors, educational status, life style, mediterranean diet, cross-sectional studies

## Abstract

Background: Obesity is a major public health concern and shows a clear social gradient, with higher prevalence among individuals with lower socioeconomic position. Educational level is a key indicator of socioeconomic status, but the extent to which lifestyle factors explain its association with obesity remains unclear. Objective: To examine the association between educational level and obesity in a working-age population and to evaluate how adjustment for lifestyle factors influences the magnitude of the association between educational level and obesity. Methods: A cross-sectional study was conducted among 3108 working-age adults undergoing occupational health assessments in Spain. Educational level was categorised into three groups (higher, intermediate, and primary or none). Obesity was defined as a body mass index ≥30 kg/m^2^. Lifestyle variables included smoking status, physical activity assessed using the International Physical Activity Questionnaire (IPAQ-SF), and adherence to the Mediterranean diet evaluated with the MEDAS-14 score. Sequential logistic regression models were used to estimate odds ratios (ORs) and 95% confidence intervals (95% CI), with progressive adjustment for demographic, behavioural, and clinical factors. Results: The overall prevalence of obesity was 16.6%, with a clear gradient across educational levels (11.5% in higher education vs. 19.8% in primary or no education). In crude analyses, individuals with the lowest educational level had higher odds of obesity (OR 1.89; 95% CI 1.46–2.45). Adjustment for age and sex attenuated the association (OR 1.72; 95% CI 1.32–2.24), with further reduction after inclusion of lifestyle factors (OR 1.63; 95% CI 1.24–2.13). In the fully adjusted model, the association remained statistically significant (OR 1.61; 95% CI 1.18–2.21), indicating that adjustment for lifestyle factors attenuated the association between educational level and obesity, although the association remained statistically significant. Conclusions: Lower educational level is associated with a higher risk of obesity. Adjustment for lifestyle factors attenuated this association, although a statistically significant relationship remained. These findings support the role of education as a fundamental determinant of health and highlight the need for strategies addressing broader social and structural determinants of obesity.

## 1. Introduction

Obesity is widely recognised as one of the major public health challenges of the 21st century, given its well-established role in the development of cardiovascular disease, type 2 diabetes, and other chronic conditions [1,2]. Despite considerable efforts to curb its prevalence, rates of obesity continue to rise in many regions, suggesting that conventional approaches focused on individual risk factors may be insufficient [3].

In this context, increasing attention has been directed towards the role of socioeconomic inequalities. A substantial body of evidence indicates that obesity does not occur randomly within populations, but rather follows a clear social gradient, with higher prevalence consistently observed among individuals with lower socioeconomic position, particularly those with lower educational attainment [4,5,6]. This pattern has been documented across different countries and age groups, pointing towards underlying structural mechanisms rather than isolated behavioural differences [5,7]. Education, as a core component of socioeconomic position, is of particular interest because it reflects not only knowledge acquisition but also long-term access to opportunities, resources, and health-related decision-making capacity [8,9]. Individuals with lower educational levels are more likely to experience adverse living conditions and may face greater barriers to adopting and maintaining healthy behaviours.

Lifestyle factors such as diet, physical activity, and smoking are commonly proposed as key pathways linking socioeconomic position to obesity [10,11,12]. Indeed, these behaviours tend to cluster within socially disadvantaged groups and are known to contribute to energy imbalance and weight gain [11,13]. However, the extent to which these factors fully account for socioeconomic differences in obesity remains open to debate. Some studies suggest that adjusting for lifestyle behaviours substantially attenuates the association between socioeconomic status and obesity, while others report that a residual effect persists even after accounting for these variables. This raises the possibility that education may influence obesity through additional pathways that are not entirely captured by conventional behavioural measures.

Clarifying this issue is not merely of academic interest. If socioeconomic inequalities in obesity are largely explained by lifestyle factors, interventions targeting behaviour change may be sufficient. On the other hand, if an association between educational level and obesity persists after adjustment for lifestyle factors, this would support the need for broader structural approaches aimed at reducing social inequalities in health [8,14].

Against this background, the present study examines the association between educational level and obesity in a working-age population and evaluates how adjustment for lifestyle factors influences the magnitude of this association. By applying sequential adjustment models, we aimed to assess changes in effect estimates after inclusion of lifestyle-related variables.

## 2. Methods

### 2.1. Study Design and Population

A cross-sectional study was conducted using data collected between January 2019 and September 2020 from a large occupational cohort of working-age adults in several Spanish autonomous communities. Participants were recruited during routine occupational health assessments performed as part of periodic workplace health surveillance programmes across different employment sectors. These assessments are routinely conducted among actively employed workers and include standardised collection of anthropometric, clinical, sociodemographic, and lifestyle information. The occupational health setting provided an opportunity to evaluate educational level, lifestyle factors, and obesity within a real-world employed population. Consequently, the study population should be considered an occupational health sample of actively employed adults rather than a population-based sample.

**Figure 1:**
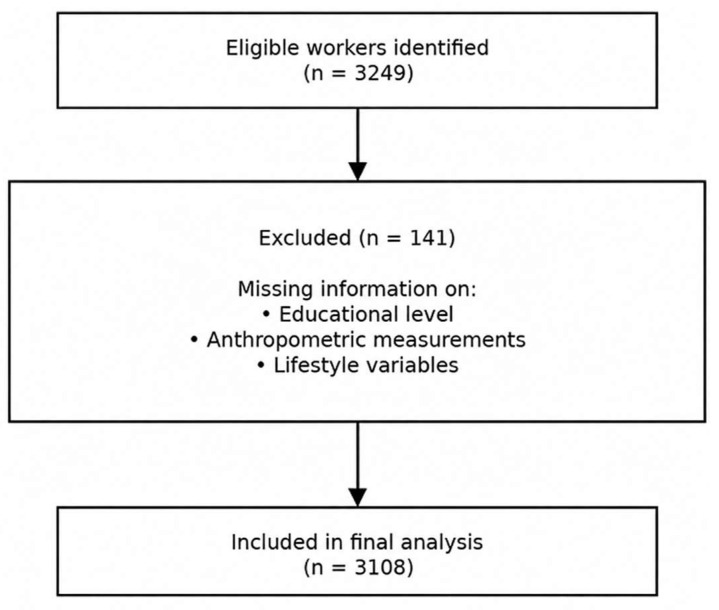
Flow diagram of participant selection.

Participants were included if complete information was available for educational level, anthropometric measurements, and lifestyle variables. Individuals with missing data in key exposure or outcome variables were excluded from the analysis. A total of 3249 eligible workers were initially identified. Of these, 141 were excluded because of missing information on educational level, anthropometric measurements, or lifestyle variables. The final analytical sample therefore consisted of 3108 participants, corresponding to a participation rate of 95.7%. The participant selection process is summarised in Figure 1. The use of occupational cohorts has been shown to provide valuable insights into cardiometabolic risk and its determinants in active populations [15,16].

### 2.2. Study Variables

#### 2.2.1. Exposure: Educational Level

Educational attainment was used as the primary indicator of socioeconomic position. Educational level was self-reported during the occupational health interview and was classified into three categories according to the Spanish educational system: higher education (university degree, postgraduate degree, or equivalent tertiary education), intermediate education (upper secondary education, vocational training, or equivalent qualifications), and primary or no formal education (primary school education, incomplete compulsory education, or no formal educational qualifications). This categorisation reflects commonly used approaches in epidemiological research and captures gradients in socioeconomic conditions across the adult population [17].

Education was selected over other socioeconomic indicators due to its relative stability over time and its strong association with health-related behaviours and long-term risk of chronic disease [18,19].

#### 2.2.2. Outcome: Obesity

The main outcome of interest was obesity, defined as a body mass index (BMI) ≥ 30 kg/m^2^, calculated from measured weight and height. Anthropometric measurements were obtained during standardised occupational health examinations by trained healthcare personnel. Body weight was measured using a calibrated Tanita BC-418 MA scale (TANITA Corporation, Tokyo, Japan), with participants wearing underwear and no shoes. Height was measured in the standing position using a SECA 220 stadiometer (SECA, Chino, CA, USA). BMI was subsequently calculated as weight in kilograms divided by height in metres squared.

BMI remains the most widely used indicator in epidemiological studies assessing obesity at the population level, despite known limitations related to body composition [20].

#### 2.2.3. Lifestyle Variables

Lifestyle factors were considered as explanatory variables potentially contributing to the association between educational level and obesity. These included smoking status, physical activity, and adherence to a Mediterranean dietary pattern, all assessed using validated instruments.

The occupational health database did not distinguish between never smokers and former smokers; therefore, a more detailed categorisation of smoking status was not possible. Participants were considered current smokers if they reported smoking tobacco regularly at the time of the assessment, regardless of the number of cigarettes consumed per day. Individuals who reported not currently smoking were classified as non-smokers, including both never smokers and former smokers [21].

Physical activity was evaluated using the short form of the International Physical Activity Questionnaire (IPAQ-SF), a widely used and validated tool for assessing physical activity in epidemiological studies [22]. The IPAQ-SF captures the frequency and duration of vigorous, moderate, and walking activities over the previous seven days. Following established IPAQ scoring guidelines, participants classified as having moderate or high physical activity levels were considered physically active, whereas those classified as having low physical activity levels were considered inactive.

Dietary habits were assessed using the 14-item Mediterranean Diet Adherence Screener (MEDAS-14), which evaluates adherence to key components of the Mediterranean diet, including consumption of olive oil, fruits, vegetables, legumes, fish, and processed foods. The MEDAS-14 score ranges from 0 to 14, with higher scores indicating greater adherence to the Mediterranean dietary pattern.

Participants were classified into two categories according to established cut-off points: low adherence (<9 points) and high adherence (≥9 points) [23]. This threshold has been widely used in Mediterranean population studies and has been shown to discriminate between lower and higher adherence to the Mediterranean dietary pattern.

The inclusion of these validated instruments allows for a more robust assessment of lifestyle behaviours and reduces the likelihood of measurement bias when evaluating their relationship with educational level and obesity.

### 2.3. Additional Covariates

Age and sex were included as core covariates in all analyses due to their strong and consistent association with obesity. Additionally, the presence of diabetes was considered a clinical variable given its close relationship with adiposity and metabolic dysfunction [24]. Diabetes status was determined from occupational health records, previously documented medical diagnoses, self-reported medical history obtained during the occupational health interview, and laboratory data available from the occupational health assessment.

### 2.4. Statistical Analysis

Descriptive analyses were performed to characterise the study population across educational categories. Continuous variables were expressed as mean and standard deviation, while categorical variables were presented as frequencies and percentages.

The distribution of continuous variables was assessed using the Kolmogorov–Smirnov test. Variables showing an approximately normal distribution were summarised as means and standard deviations (SD). Comparisons between groups were performed using Student’s *t*-test for independent samples.

Prior to fitting the regression models, multicollinearity among the independent variables was assessed using variance inflation factors (VIFs). All VIF values were below 10, indicating the absence of problematic multicollinearity among the variables included in the models.

The association between educational level and obesity was evaluated using logistic regression models. To assess how adjustment for lifestyle factors influenced the association between educational level and obesity, a sequential modelling approach was applied:

- Model 1: crude association
- Model 2: adjusted for age and sex
- Model 3: additionally adjusted for lifestyle factors (smoking, physical activity, diet)
- Model 4: sensitivity analysis additionally adjusted for diabetes.

Changes in effect estimates across models were examined to evaluate how adjustment for lifestyle variables influenced the magnitude of the association between educational level and obesity. The percentage attenuation of the odds ratios after inclusion of lifestyle factors was calculated as a descriptive measure of changes in the strength of the association across sequential models. Percentage attenuation was calculated as [(ORcrude − ORadjusted)/ORcrude] × 100, where ORcrude represents the odds ratio from the unadjusted model and ORadjusted represents the odds ratio after adjustment. This approach was not intended to estimate direct or indirect effects and should not be interpreted as a formal mediation analysis. These analyses should not be interpreted as formal mediation analyses or as evidence of direct and indirect causal effects, a commonly used approach in epidemiological studies when formal causal mediation methods are not applied [25,26]. Given the cross-sectional design, temporal ordering among educational level, lifestyle behaviours, and obesity could not be established.

Results were expressed as odds ratios (ORs) with 95% confidence intervals (95% CI). A two-sided *p*-value < 0.05 was considered statistically significant.

All analyses were conducted using IBM SPSS Statistics version 30.0 (IBM Corp., Armonk, NY, USA).

## 3. Results

### 3.1. Study Population

A total of 3108 participants were included in the analysis. The mean age of the study population was 42.7 years (SD 9.4), and 40.7% were women. Educational level showed a clear gradient across several baseline characteristics, with individuals in lower educational categories tending to be older and more frequently exposed to adverse behavioural and clinical profiles.

The main characteristics of the study population according to educational level are presented in Table 1.

In particular, lower educational attainment was associated with a higher prevalence of smoking and physical inactivity, as well as a greater proportion of individuals with diabetes, suggesting a clustering of behavioural and metabolic risk factors within socially disadvantaged groups.

### 3.2. Prevalence of Obesity Across Educational Levels

The overall prevalence of obesity in the study population was 16.6%. A clear and progressive gradient was observed across educational categories, with higher prevalence among individuals with lower educational attainment.

Specifically, obesity prevalence increased from 11.5% in participants with higher education to 19.8% among those with primary or no formal education, with intermediate values observed in the middle category. This gradient was statistically significant and consistent across the distribution, indicating a robust association between educational level and obesity.

### 3.3. Association Between Educational Level and Obesity

To further explore the relationship between educational level and obesity, sequential logistic regression models were fitted, progressively adjusting for demographic, behavioural, and clinical factors. The results are summarised in Table 2.

As shown in Table 2, a lower educational level was associated with higher odds of obesity in crude analyses, with a clear gradient across categories. Adjustment for age and sex led to a modest attenuation of the estimates, although the association remained statistically significant for individuals with primary or no formal education.

Further adjustment for lifestyle factors, including smoking, physical activity, and dietary habits, resulted in an additional reduction in the magnitude of the association. This pattern indicates that adjustment for behavioural factors attenuated the association between educational level and obesity.

In a sensitivity analysis additionally adjusted for diabetes, individuals with the lowest educational level continued to exhibit significantly higher odds of obesity (OR 1.61, 95% CI 1.18–2.21), indicating that the observed association was robust to this additional adjustment.

**Figure 2:**
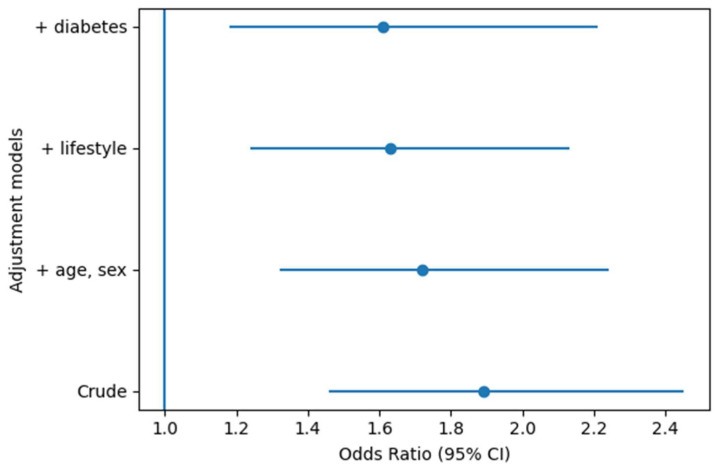
Association between educational level and obesity across sequential models.

The progressive attenuation of the association across models is further illustrated in Figure 2.

Figure 2 shows a gradual reduction in the strength of the association after adjustment for lifestyle and clinical factors, while the association remained statistically significant across models.

To further quantify the contribution of lifestyle factors to the association between educational level and obesity, changes in the effect estimates across sequential models were examined. These results are presented in Table 3.

Percentages represent descriptive changes in effect estimates relative to the crude model and should not be interpreted as estimates of mediation, direct effects, or indirect effects.

As shown in Table 3, the inclusion of demographic variables resulted in a modest reduction in the strength of the association. Further adjustment for lifestyle factors led to an additional attenuation, indicating that adjustment for smoking, physical activity, and diet attenuated the association between educational level and obesity.

## 4. Discussion

In this cross-sectional study of working-age adults, a lower educational level was consistently associated with a higher prevalence and increased odds of obesity. The observed gradient was robust across analytical approaches and persisted after adjustment for demographic, behavioural, and clinical factors. Although the inclusion of lifestyle variables led to a modest attenuation of the association, a statistically significant association remained after adjustment.

Taken together, these findings indicate that adjustment for behavioural factors attenuated, but did not eliminate, the association between educational level and obesity. Rather than representing isolated individual choices, obesity appears to reflect a broader social pattern shaped by both behavioural and structural determinants.

The presence of a clear educational gradient in obesity observed in this study is consistent with a large body of epidemiological evidence. Previous research has repeatedly shown that obesity is disproportionately concentrated among individuals with lower socioeconomic position, particularly when measured through educational attainment [27,28]. This pattern has been documented across different geographical settings and remains evident even in countries with universal healthcare systems, suggesting that access to care alone does not eliminate these disparities.

Importantly, our findings are in line with studies indicating that adjustment for lifestyle behaviours only partially reduces socioeconomic differences in obesity. For instance, analyses conducted in European cohorts have demonstrated that although diet and physical activity are socially patterned, their contribution to explaining inequalities in obesity is limited [29,30]. Similarly, longitudinal studies have reported that socioeconomic gradients in body mass index persist over time despite accounting for behavioural factors, reinforcing the notion that additional mechanisms are involved [31].

Taken together, these observations support the interpretation that educational inequalities in obesity cannot be fully attributed to differences in individual behaviour, and instead reflect more complex and multifactorial processes.

One of the most relevant aspects of the present study is the relatively modest attenuation of the association between educational level and obesity after adjustment for lifestyle variables. While behaviours such as physical inactivity, smoking, and dietary patterns clearly contribute to obesity risk, the limited reduction in effect estimates suggests that these factors capture only part of the underlying pathways.

This finding is consistent with the concept of socioeconomic position as an upstream determinant of health. Educational level has been associated with obesity through a wide range of mechanisms proposed in previous research, including early-life exposures, cumulative disadvantage, and differential access to material and social resources across the life course [32,33]. From this perspective, lifestyle behaviours may reflect broader social conditions and could contribute to the observed association between educational level and obesity.

Moreover, the use of self-reported lifestyle variables may underestimate their true contribution, particularly in socially patterned behaviours such as diet and physical activity. Nevertheless, even accounting for potential measurement error, the persistence of the association suggests that important determinants remain unmeasured. This interpretation is consistent with recent evidence from formal mediation analyses. Bartoskova Polcrova et al. demonstrated that educational inequalities in adiposity are influenced by multiple pathways, including lifestyle, socioeconomic, and mental health factors, and that the relative contribution of these pathways may differ by sex. Their findings highlight the added value of formal mediation approaches for disentangling direct and indirect mechanisms and reinforce the distinction between attenuation of associations after adjustment and causal mediation effects [34].

Although environmental and contextual factors were not directly measured in the present study, previous research has highlighted their potential importance in shaping obesity risk. Previous studies have suggested that individuals with lower educational attainment may be more likely to be exposed to environments characterised by limited access to healthy foods, reduced opportunities for physical activity, and greater availability of energy-dense, nutrient-poor options [35,36].

In addition to material conditions, previous research has suggested that psychosocial factors may also contribute to obesity risk. Chronic exposure to stress associated with socioeconomic disadvantage has been linked to dysregulation of neuroendocrine pathways, including activation of the hypothalamic–pituitary–adrenal axis, which may contribute to weight gain and metabolic disturbances [37,38]. Recent evidence has also highlighted the influence of social determinants and stress on dietary behaviours, reinforcing the notion that lifestyle factors are shaped by broader socioeconomic and psychosocial conditions rather than operating in isolation [39]. These mechanisms have been proposed as potential explanations for the persistence of socioeconomic inequalities in obesity beyond behavioural factors. However, these pathways were not directly measured in the present study and therefore cannot be evaluated using our data.

Taken together, evidence from previous studies suggests that obesity may be influenced by a broader social and environmental context, rather than solely by individual lifestyle choices. However, these factors were not directly assessed in the present study and should therefore be considered as potential explanatory mechanisms rather than findings derived from our data.

### 4.1. Strengths and Limitations

This study has several strengths that should be considered. It includes a relatively large sample of working-age adults, with objectively measured anthropometric data and the use of validated instruments to assess key lifestyle behaviours. The application of sequential regression models allowed for a structured assessment of the contribution of different factors to the observed association.

At the same time, several limitations should be acknowledged. The cross-sectional design precludes causal inference, and reverse causation cannot be ruled out. Lifestyle variables were self-reported and may be subject to recall or reporting bias. Additionally, although major behavioural factors were included, other relevant determinants such as environmental exposures, income, or psychosocial stress were not directly measured.

Because diabetes may lie on pathways linking socioeconomic factors and obesity, the model including diabetes should be interpreted as a sensitivity analysis rather than a primary adjustment model.

An additional limitation relates to the occupational nature of the study population. Participants were recruited during routine occupational health assessments and therefore represent an actively employed population. Consequently, unemployed individuals, retired adults, persons outside the labour market, and individuals with severe illness were not represented. This may have introduced a healthy worker effect, whereby employed individuals tend to have a more favourable health profile than the general population. Therefore, caution is warranted when generalising these findings beyond working-age employed adults.

Accordingly, the study sample should not be considered fully representative of the general working-age population.

The lifestyle variables were analysed using categorised measures available in the study database. Therefore, more granular classifications of smoking status, physical activity, or Mediterranean diet adherence, as well as sensitivity analyses using continuous lifestyle scores, could not be performed. This categorisation may have led to some loss of information and residual confounding.

Furthermore, smoking status was available only as current smoker or non-smoker, preventing differentiation between never smokers and former smokers and potentially introducing residual confounding.

These limitations should be considered when interpreting the results, although they are unlikely to fully explain the observed associations.

### 4.2. Public Health Implications

The findings of this study have important implications for the design of public health strategies aimed at reducing obesity. Interventions focusing exclusively on individual behaviour change may have limited effectiveness if broader social determinants are not addressed.

Policies aimed at reducing educational inequalities, improving access to healthy environments, and addressing structural barriers to healthy living have been proposed as potential strategies to address obesity-related inequalities in previous research. This aligns with current public health frameworks that emphasise the importance of upstream interventions targeting the social determinants of health.

## 5. Conclusions

Lower educational level was associated with higher odds of prevalent obesity in this working-age population. Sequential adjustment for lifestyle factors modestly attenuated this association, suggesting that these behaviours may contribute to, but do not fully account for, educational inequalities in obesity. A statistically significant association remained after adjustment, indicating that additional factors may be involved. These findings should be interpreted within the context of an employed working-age population and may not be directly generalisable to the broader adult population.

## Figures and Tables

**Table 1:** Characteristics of the study population according to educational level.

Variable	Higher Education (*n* = 732)	Intermediate (*n* = 760)	Primary/None (*n* = 1616)	*p*-Value
Age, mean (SD)	35.8 (8.2)	44.6 (8.9)	45.0 (9.8)	<0.001
Female, *n* (%)	348 (47.5)	300 (39.5)	617 (38.2)	<0.001
Obesity, *n* (%)	84 (11.5)	112 (14.7)	320 (19.8)	<0.001
Current smokers, *n* (%)	204 (27.9)	208 (27.4)	575 (35.6)	<0.001
Physical inactivity, *n* (%)	248 (33.9)	280 (36.8)	667 (41.3)	0.002
Low adherence to Mediterranean diet (MEDAS-14), *n* (%)	238 (32.5)	312 (41.1)	792 (49.0)	<0.001
Diabetes, *n* (%)	4 (0.5)	12 (1.6)	56 (3.5)	<0.001

**Table 2:** Association between educational level and obesity: sequential logistic regression models.

Model	Intermediate vs. Higher OR (95% CI)	Primary vs. Higher OR (95% CI)
Model 1 (crude)	1.33 (1.01–1.75)	1.89 (1.46–2.45)
Model 2 (+age, sex)	1.28 (0.97–1.68)	1.72 (1.32–2.24)
Model 3 (+lifestyle factors)	1.22 (0.92–1.60)	1.63 (1.24–2.13)
Model 4 (sensitivity analysis: +diabetes)	1.22 (0.88–1.69)	1.61 (1.18–2.21)

**Table 3:** Descriptive attenuation of odds ratios across sequentially adjusted models.

Model	OR (Primary vs. Higher)	% Change
Crude	1.89	—
+age, sex	1.72	−9%
+lifestyle	1.63	−14%
Fully adjusted	1.61	−15%

## Data Availability

The datasets generated and analyzed during the current study are maintained in a secure institutional repository at ADEMA University School. Access to fully anonymised data may be considered upon reasonable request to the corresponding author, subject to compliance with applicable ethical requirements and current data protection legislation. The data presented in this study are available on request from the corresponding author. Data are available from ADEMA University School upon reasonable request, subject to data protection re-strictions.
